# Exploring the Role of Intracorporeal Ultrasound in Partial Nephrectomies: A Systematic Review

**DOI:** 10.7759/cureus.73293

**Published:** 2024-11-08

**Authors:** Mohamed S Mohsin, Rebecca Jess, Habeeb Abdulrasheed, Humood Almedej, Banan Osman, Nader Gaballa, Shankar Chandrasekharan

**Affiliations:** 1 Department of Urology, University Hospitals Birmingham, Birmingham, GBR

**Keywords:** intraoperative ultrasound, laparoscopic procedure, partial nephrectomy, renal cell carcinoma (rcc), robot-assisted partial nephrectomy, uro-oncology

## Abstract

Renal cell carcinoma accounts for the sixth most common cancer in the United Kingdom. With the increasing application of cross-sectional imaging, the frequency of incidental renal masses has increased over time. Laparoscopic and robot-assisted partial nephrectomy has become the standard of care in the management of size and stage-appropriate renal masses. The objective of this systematic review was to analyse the surgical outcomes when intracorporeal ultrasound was utilised as an adjunct in partial nephrectomy.

A comprehensive search in PubMed and Google Scholar was performed in July 2024 for publications in the English language. The primary endpoint was to evaluate the role of intracorporeal ultrasound as an adjunct in robotic partial nephrectomies and its impact on tumour clearance.

After identifying 609 records, 52 records were screened and 44 records were sought for retrieval. Eight publications were included in this systematic review comprising 765 patients. Seven of the eight studies reported outcomes from single centres. The mean percentage of negative surgical margins was 97.6% (range = 92.1-100%).

The use of intracorporeal ultrasound as an adjunct in partial nephrectomy for T1 renal cell cancer has proven to improve the rates of negative surgical margins thereby reducing the incidence of local recurrence and distant metastasis.

## Introduction and background

Renal cell carcinoma (RCC) is the most common form of kidney cancer accounting for 90% of all renal malignancies [1]. In the United Kingdom (UK), kidney cancer is the sixth most common cancer accounting for 4% of all new cancer diagnoses. Of all cases that are diagnosed, 34% are in patients aged 75 years and older [2]. The overall incidence of kidney cancer is more common amongst men with 66% of all RCCs being diagnosed in males [3].

In terms of mortality, RCC represents the single urological malignancy with the highest mortality rate owing to the rate at which the disease progresses with five-year survival rates in metastatic RCC being 12% [4].

With the increasing use of cross-sectional imaging, a rise in incidental detection of RCC has been reported [1,3,5], accounting for the increased incidence of kidney cancer. This is demonstrated by UK-specific age-standardised incidence rates for both genders combined, growing by 92% over 26 years (data points: 1993-1995 and 2017-2019). More than 40% of all new cancers meet the criteria for classification as small renal masses (SRMs), which are defined as renal masses of 4 cm or less [6,7]. Notably, while up to 80% of all SRMs are malignant, up to 17% of patients with incidental RCC also have distant metastasis at the point of diagnostic and staging investigations [3].

The treatment options for RCC include conservative management, focal therapy, surgery, and systematic therapy/immunotherapy in metastatic disease. Surgical options include the use of open/laparoscopic/hand-assisted and robotic techniques to achieve a partial nephrectomy (PN) also referred to as nephron-sparing surgery or radical nephrectomy (RN). With the advances in robotics and its widespread application amongst centres worldwide, a general trend towards robotic nephrectomy is becoming apparent.

Indications for PN include bilateral renal tumours and hereditary RCCs, a baseline of renal impairment or an impaired unaffected kidney with the risk of further deterioration following an RN, a tumour in a solitary kidney, and a single tumour measuring 4 cm or less (T1a) [8]. They can be further classified into absolute, relative, and elective indications [9].

The problem statement concerns the incidence rates of positive surgical margins (PSMs) following PN and its implications on cancer-specific outcomes. A PSM is considered an extension of the RCC, which includes and extends beyond the limits or margins of the excised specimen [10]. A negative surgical margin (NSM) is considered as the histopathological limits of the RCC being within the limits or margins of the excised specimen. PSMs and NSMs can only be confirmed based on histological analysis of the specimen.

A systematic review investigating surgical margins after PN in pT1 RCCs as a prognostic factor for the risk of local recurrence (LR) in pT1 RCC, including eight studies, found that up to 34.4% of all cases included had PSM [11]. In the same study, up to 9.1% of patients with a PSM had LR compared to 1.5% of patients with NSMs.

The purpose of this study was to conduct a systematic review of current evidence on the utility of intracorporeal ultrasound (InUS) as an adjunct in robotic partial nephrectomies and its impact on tumour clearance to determine if InUS can be leveraged to improve oncological outcomes.

## Review

Methodology

During July 2024, PubMed and Google Scholar databases were searched separately without date restriction using the following terms: “intracorporeal”, ”ultrasound”, and “nephrectomy”. Inclusion criteria were as follows: patients > 18 years old; series pertaining to T1 renal tumours managed by laparoscopic partial nephrectomy (LPN) or robot-assisted partial nephrectomy (RAPN); prospective studies, retrospective studies, multi-centre studies, or single studies.

Exclusion criteria were studies in languages other than English with no direct translation, irrelevant to the systematic review, inaccessible full texts, animal studies, RCC in the paediatric population, systematic reviews, book chapters, video abstracts, limited case reports or series, abstracts, studies related predominantly to T2 tumours, studies assessing novel techniques and technology, studies combining other treatment modalities with PN, studies utilising frozen section, comparative studies, and those with fewer than 20 patients.

**Figure 1:**
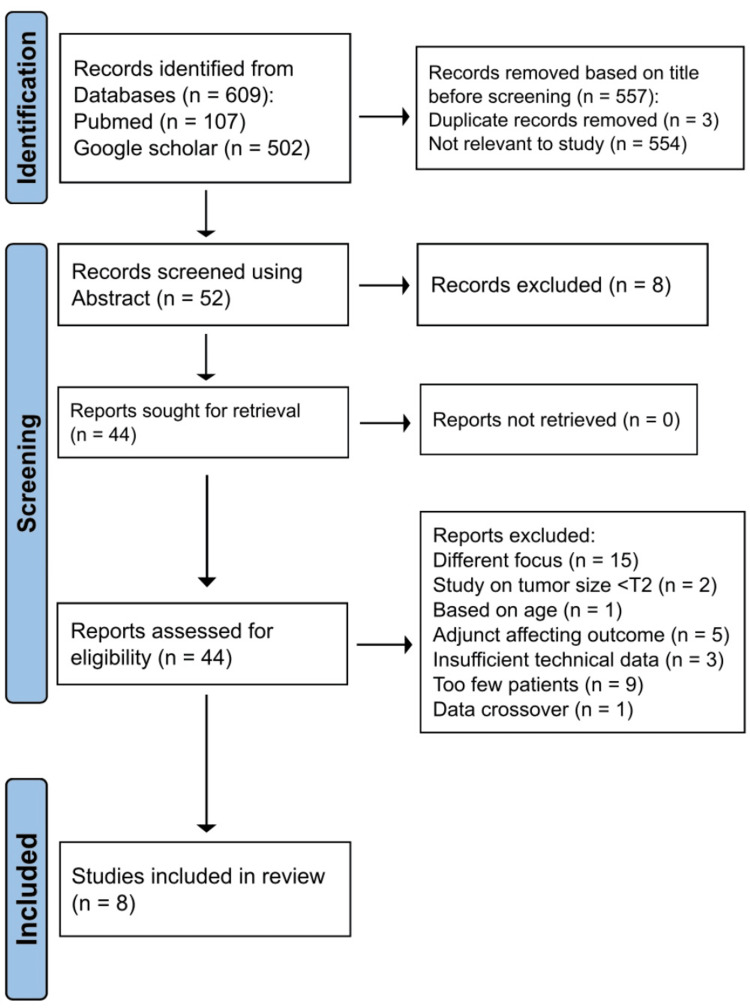
Preferred Reporting Items for Systematic Reviews and Meta-Analyses (PRISMA) flow diagram.

This systematic review was conducted in accordance with the Preferred Reporting Items for Systematic Reviews and Meta-Analyses (PRISMA) guidelines (Figure 1) [12]. The patients, intervention, comparison, and outcomes (PICO) method was used to develop the hypothesis. Patients: cT1 RCC; intervention: patients who underwent PN with the use of InUS; comparison: PN without the use of InUS; outcome: incidence of PSM and NSM on final pathology.

Where overlap in multiple publications from single centres was found, studies were scrutinised to avoid crossover and contamination of data. Abstracts and full-text articles were independently reviewed by MM and HA. A risk of bias assessment was also carried out by two independent reviewers (RJ and HA) using the Newcastle-Ottawa Scale for observational studies (Table 1) [13].

**Table 1:** Newcastle-Ottawa Scale scores for observational studies. Newcastle-Ottawa Scale [13].

Authors	Selection (n/4)	Comparability (n/2)	Outcome (n/3)	Total (n/9)
Autorino et al. [14]	4	2	3	9
Chung et al. [15]	3	2	2	7
Gill et al. [16]	3	2	3	8
Hu et al. [17]	3	2	3	8
Patel et al. [18]	4	2	3	9
Sun et al. [19]	3	2	3	8
Yang et al. [20]	3	2	3	8
Zeccolini et al. [21]	3	2	3	8

Results

A total of 765 patients were included across eight studies from three countries (USA, Italy, and China), and nine centres. The mean patient age was 58.8 years and the mean tumour size for all renal lesions was 3.04 (range = 0.7-7.9 cm). The patient and tumour characteristics, including the RENAL Nephrometry Score [22] and PADUA (Preoperative Aspects and Dimensions Used for an Anatomical classification of renal tumours) score [23] where applicable, have been detailed in Table 2.

**Table 2:** Description of the study, patient, and tumour characteristics. NS: not specified; N/A: not applicable; SC: single centre; MC: multi-centre; RENAL score: Radius, Exophytic/endophytic, Nearness to the collecting system or sinus, Anterior/posterior descriptor, and Location relative to the polar line; PADUA: Preoperative Aspects and Dimensions Used for an Anatomical classification of renal tumours; cm: centimetres. RENAL Nephrometry Score: Kutikov & Uzzo (2009) [22]. PADUA score: Ficarra et al. (2009) [23].

Authors	Year	Country	Centre (SC/MC)	Subgroup (where applicable)	Number of patients (n)	Mean age (years)	Age range (years)	Mean tumour size (cm)	Tumour size range (cm)	RENAL score	PADUA score
Autorino et al. [14]	2014	USA	SC	N/A	65	56	NS	2.6	NS	Low (n = 6), moderate (n = 36), high (n = 23)	NS
Chung et al. [15]	2011	USA	SC	N/A	55	57.8	32-83	2.3	1-4.5	NS	NS
Gill et al. [16]	2002	USA	SC	N/A	50	64	30-87	3	1.4-7	NS	NS
Hu et al. [17]	2014	USA	MC	N/A	227	60	52-66	2.3	1.7–3.1	Low (n = 13), moderate (n = 117), high (n = 39), NS (n = 58)	NS
Patel et al. [18]	2009	USA	SC	Tumour > 4 cm	15	59	44-76	5	4.1-7.9	NS	NS
Patel et al. [18]	2009	USA	SC	Tumour ≤ 4 cm	56	60.1	35-81	2.5	0.7-3.8	NS	NS
Sun et al. [19]	2021	China	SC	N/A	38	50.7	NS	2.9	NS	Low (n = 6), moderate (n = 27), high (n = 5)	Low (n = 0), moderate (n = 12), high (n = 26)
Yang et al. [20]	2019	China	SC	N/A	199	53.4	NS	3.6	NS	6.6 (SD = 1.7)	NS
Zeccolini et al. [21]	2015	Italy	SC	N/A	60	63	44-80	3.2	2-6.7	NS	NS

The results of the technique, approach, transducer used, final histological outcome, and rates of PSM and NSM have been detailed in Table 3.

**Table 3:** Description of technique, approach, transducer, final histological outcome, and rates of R0 and R1 margins. CS: collecting system; TP: transperitoneal; RP: retroperitoneal; PSM: positive surgical margin; NSM: negative surgical margin; NS: not specified; RAPN: robotic-assisted partial nephrectomy; LPN: laparoscopic partial nephrectomy; RCC: renal cell carcinoma; OC: oncocytoma; AML: angiomyolipoma; SC: single centre; MC: Multi-centre; US: ultrasound; InUS: intracorporeal ultrasound.

Authors	CS involvement (n)	Proximity to CS	Location in parenchyma (>50% exophytic, <50% exophytic, endophytic)	Technique	TP (n)	RP (n)	Transducer	Histology	NSM (%)	PSM (%)	Note
Autorino et al. [14]	NS	NS	Endophytic	RAPN	65	0	Laparoscopic ultrasound probe and drop in flexible US probe	RCC (n = 48), OC (n = 5), AML (n = 3), cyst (n = 7), other (n = 2)	95.4	4.6	Included 2 patients with 3a RCC on histology
Chung et al. [15]	NS	NS	Endophytic	LPN	48	7	Not specified	RCC (n = 40), OC (n = 6), AML (n = 5), cyst (n = 4)	100	0	SC experience in LPN for endophytic tumours
Gill et al. [16]	NS	NS	>50% exophytic	LPN	28	22	Laparoscopic flexible and steerable colour Doppler probe via a 10 or 12 mm port	RCC (n = 34), OC (n = 5), AML (n = 8), other (n = 3)	100	0	SC experience in LPN for exophytic tumours
Hu et al. [17]	NS	≥7 mm (n = 60), >4 cm (n = 37), ≤4 cm (n = 91), NS (n = 39)	>50% exophytic (n = 78), <50% exophytic (n = 78), endophytic (n = 33), NS (n = 38)	RAPN	0	227	Laparoscopic ultrasound probe	RCC (n = 179), unclassified (n = 2), benign (n = 45)	96.5	3.5	MC experience in retroperitoneal RAPN
Patel et al. [18]	10 abutting collecting system	NS	>50% exophytic (n = 11), <50% exophytic (n = 2)	RAPN	15	0	Laparoscopic ultrasound probe	RCC (n = 10), OC (n = 1), AML (n = 3), benign (n = 1)	100	0	SC experience in RAPN. Grouped into ≤4 cm and >4 cm
Patel et al. [18]	31 abutting collecting system	NS	>50% exophytic (n = 28), <50% exophytic (n = 19), endophytic (n = 5)	RAPN	56	0	Laparoscopic ultrasound probe	RCC (n = 41), OC (n = 4), AML (n = 6), benign (n = 5)	94.7	5.3	
Sun et al. [19]	13	NS	Endophytic	RAPN	NS	NS	BK Medical 8826	RCC (n = 32)	92.1	7.9	SC experience in the application of InUS for RAPN for endophytic tumours. 15% PSM margins were noted in the non-InUS group.
Yang et al. [20]	NS	NS	NS	LPN	0	199	Flexible US probe UST-5536-7.5 Ultrasound, Aloka	RCC (n = 169), OC (n = 4), AML (n = 23), cyst (n = 3), other (n = 2)	100	0	SC experience in the application of InUS in RP LPN.
Zeccolini et al. [21]	NS	NS	NS	RAPN	60	0	Laparoscopic ultrasound probe	RCC (n = 58), OC (n = 1), AML (n = 1)	100	0	SC experience in RAPN.

The technique for PN with US guidance included laparoscopic PN in three studies and robotic-assisted PN in five studies with a higher proportion of patients undergoing a retroperitoneal (455 patients) compared to a transperitoneal approach (272 patients).

Six of the eight studies reported the location of the tumour in relation to the parenchyma. A total of 167 cases were predominantly exophytic with >50% of the tumour volume being exophytic; 99 cases had <50% of tumour volume being exophytic and 196 cases had endophytic tumours. The location of the tumour in relation to the parenchyma was not specified in 303 cases.

The histology reported from each study has been provided in Table 3. In summary, RCC accounted for 611 reports, oncocytoma in 26 specimens, angiomyolipoma in 49 specimens, and “other pathology”, including benign lesions and cysts, in 74 specimens. The mean proportion of NSMs was 97.6% (range = 92.1%-100%) while the mean proportion of PSMs was 2.36%% (range = 0%-7.9%).

Reviewers also determined that the median Newcastle-Ottawa Scale score [11] for observational studies was 8 (range = 7-9; Table 1), showing that all studies used had a low risk of bias and were of good quality.

Discussion

Kidney cancer is the 14th most common cancer worldwide and the 16th most common cause of cancer-related death with the incidence being highest in Asia, followed by Europe and North America [24]. Considering cancer-specific mortality rates, a study [25] conducted in the United States of America (USA) on 165,969 patients with RCC found that 36.3% of all patients (60,290) died during follow-up with 51.3% of deaths related to kidney cancer and 37.6% related to non-cancer specific deaths. Similarly, a study conducted in China [26], which included 68,612 patients, found that the highest proportion of deaths (39.0%) occurring in years one to five following a diagnosis of RCC was cancer-specific deaths, followed by cardiovascular disease.

The most commonly occurring histological subtype is clear cell RCC accounting for 75% of all RCCs, followed by papillary RCC (10% incidence rate), chromophobe RCC (5% incidence rate), and the remainder comprised of rare variants, including medullary RCC [3].

The 2017 TNM (tumour, node, and metastasis) classification of RCC outlined in Table 4 describes the tumour characteristics in terms of tumour size and invasion of adjacent structures including Gerota’s fascia (T stage) in addition to nodal involvement (N stage) and distant metastasis (M stage) [27].

**Table 4:** Description of the TNM classification applied to RCCs. TNM: tumour characteristics, nodal involvement, and distant metastasis; RCC: renal cell carcinoma.

TNM staging	Description
Tumour characteristics	
Tx	Primary tumour cannot be assessed
T0	No evidence of a primary tumour
T1	Tumour ≤ 7 cm or less in greatest dimension, limited to the kidney
T1a	Tumour ≤ 4 cm or less
T1b	Tumour > 4 cm but ≤ 7 cm
T2	Tumour > 7 cm in greatest dimension, limited to the kidney
T2a	Tumour > 7 cm but ≤ 10 cm
T2b	Tumour > 10 cm, limited to the kidney
T3	Tumour extends into major veins or perinephric tissues but not into the ipsilateral adrenal gland and not beyond Gerota fascia
T3a	Tumour extends into the renal vein or its segmental branches or invades the pelvicalyceal system or invades perirenal and/or renal sinus fat, but not beyond Gerota fascia
T3b	Tumour grossly extends into the vena cava below the diaphragm
T3c	Tumour grossly extends into the vena cava above the diaphragm or invades the wall of the vena cava
T4	Tumour invades beyond Gerota fascia (including contiguous extension into the ipsilateral adrenal gland)
Nodal status	
Nx	Regional lymph nodes cannot be assessed
N0	No regional lymph node metastasis
N1	Metastasis in regional lymph node(s)
Metastatic status	
M0	No distant metastasis
M1	Distant metastasis

Described broadly, the treatment options for RCC require a patient and pathology-focused approach and include active surveillance of the renal mass with serial cross-sectional imaging in elderly/unfit patients or those who opt for conservative management after considering the risks and benefits. Radiofrequency ablation and cryo-ablation describe the management of renal masses by interventional radiologists utilising radiologically guided delivery of thermal energy or freezing tissue to induce cellular death [28], which can be viewed as the focal therapy counterpart of high-frequency ultrasound ablation (HIFU) used in prostate cancer.

PN, also referred to as “nephron-sparing surgery”, aims to excise the tumour in toto to maximise the preservation of uninvolved renal parenchyma, thereby mitigating the loss of renal function while minimising urological complications. These three key outcomes are known as the “trifecta” in PN [29]. In contrast, an RN includes the removal of the affected kidney, surrounding fat, and local/regional lymph nodes.

Based on data from the British Association of Urological Surgeons (BAUS), there was an increase in the median number of robotic-assisted nephrectomies from seven cases in 2012 (range = 1-45) to 12 cases in 2013 (range = 1-64) per National Health Service (NHS) trust with the uptake of the robotic platform expanding from 18 to 21 NHS trusts over the same period [30]. Data from a study looking at the uptake of surgical robots across 149 NHS trusts in the UK in 2021 confirmed that 61 robots were distributed between 48 trusts. The key findings were that the number of urological surgeries increased from 2181 per year in 2013 to 8460 per year in 2019. Robotics in urology accounted for 84.2% of all robotic procedures [31]. These data confirm the increasing adoption of the robotic platform in the UK.

In addition to the ergonomic edge offered to the surgeon, key advantages of the robotic platform include the three-dimensional depth of vision unlike the two dimensions of traditional laparoscopy and the precision of control as well as wrist articulation allowed with the robotic arm and instruments [21] as well as what is considered a shorter learning curve [32]. The drawbacks in contrast include the additional space required for docking and the console as well as the financial investment into the robotic platform and training surgeons in robotic surgery.

Considering T1a RCCs, a prematurely closed randomised control trial over 10 years, including 541 patients with solitary tumours measuring 5 cm or less, randomised to partial nephrectomy and radical nephrectomy found that the number of cancer progressions and cancer-specific deaths from renal cancer did not explain an overall survival (OS) benefit between PN and RN [33]. The value of PN in T1a tumours has been established across literature and is routine practice worldwide, including the authors’ centre. However, T1b RCCs are always the subject of careful patient selection and consideration of OS and oncological outcomes. Evidence from a multicentre study of 1454 patients undergoing PN (n = 379) or RN (n = 1075) for T1N0M0 renal cancer, including 65 and 576 patients in the T1b group for PN and RN, respectively, found no statistically significant difference in recurrence rates or cancer-specific deaths between the two cohorts, suggesting that PN can be expanded to include carefully selected RCCs of up to 7 cm [34].

The benefits of PN extend beyond cancer-specific outcomes as evidence demonstrates that the risk of postoperative chronic kidney disease (CKD) as well as cardiovascular events are lower in the PN than the RN albeit with no observable benefit in advanced CKD [35].

A meta-analysis including 39 studies (21,461 patients) showed that there was a statistically significant difference in tumour recurrence (p < 0.00001) and metastatic rates (<0.00001) in the cohort of patients with a PSM compared to patients with an NSM with no significant difference in cancer-specific death rates (CSDR) (p = 0.99) or overall death rate (ODR) from all causes (p = 0.13) [36]. One retrospective, single-centre study including 388 patients concluded that the recurrence rates and requirement to proceed to radical nephrectomy were higher in the cohort of patients with PSM with no impact on OS noted [37]. A second study including 733 patients over 15 years in a single centre analysed retrospectively showed that 6.2% and 2.5% of patients from the PSM cohort developed LR and metastasis, respectively, with no LR or metastasis noted in the NSM. This was associated with 100% recurrence-free survival and 100% metastasis-free survival in the NSM cohort, albeit there were no OS differences between the two groups (p = 0.68) [10].

Presently, there is no set guidance regarding the ideal surgical margin (SM) of safety to excise in PN. Historically, an SM of 1 cm was recommended as a margin of safety [38]. However, while the outcome of one study found that a margin of 5 mm or less would be adequate [39], two further studies found that even a margin of less than 1 mm is sufficient, provided that the entirety of the tumour has been excised [40,41].

Current suggestions to minimise the rates of PSMs include the use of cold scissors, enucleation of the tumour, hilar clamping, and InUS [40]. However, the use of the robotic platform has proven to also contribute towards lowering the incidence of PSMs as evidenced by 5.5% of patients and 12.2% of patients undergoing robot-assisted partial nephrectomy and conventional techniques, respectively, having PSMs [42]. Furthermore, while not the focus of this systematic review, “frozen sections” whereby the SM is examined for the presence of cancer cells indicating microscopic clearance can also be utilised to further reduce the incidence of PSM.

A systematic review investigating surgical margins after partial nephrectomy in pT1 RCCs as a prognostic factor for the risk of LR in pT1 RCC including eight studies found that up to 34.4% of all 3902 cases included had PSM [11] and did not report the use of InUS. In contrast, the reported rate of PSM from our systematic review of PN using InUS reported a PSM incidence of 2.36% (range: 0%-7.9%) in 765 cases. While our systematic review can be considered underpowered in comparison owing to the lack of published data, the difference in rates of PSMs can be considered significant.

The technique to fully utilise InUS is described in the literature. The principles are to first map the tumour limits or boundaries with an appreciation of proximity to the collecting system and vasculature, following which electrocautery is used to mark or “score” the margin of resection, which must include the appropriate SM to ensure resection of the entirety of the tumour [43,44]. Chung et al. [15] describe a technique of re-scanning the cauterised margin whereby the air trapped creates an acoustic shadow allowing for the reassessment of SM. This technique acts as a decision aid for the surgeon and allows for a revision of the margin with diathermy before resection.

A notable technique developed by Doerfler et al. [45] albeit limited to 12 cases expands on the use of InUS whereby an endoscopic specimen retrieval bag containing the resected tumour is filled with saline. The US probe is then inserted into the endobag to assess the pseudocapsule. If there is a grossly identifiable positive margin on the ultrasound scan, a further margin of renal parenchyma can be excised to ensure completion.

Ultrasound probes for partial nephrectomy operate at 7.5-10 Hz allowing the detection of tumours up to 4 cm in size [46]. For robot-assisted partial nephrectomy, commercially available flexible ultrasound probes have been developed for use with robotic grasping forceps. While this list is not exhaustive and the authors have no incentives or financial disclosures with any of the companies, examples include the 4-13 MHz linear array probe from Hitachi-Aloka (Tokyo, Japan), the 5-12 MHz transducer from BK Medical (Burlington, MA) as well as the 3-15 MHz transducer from Fujifilm Healthcare (Tokyo, Japan). The probes are inserted via a 10 or 12-mm port and have been engineered to allow for fine control through the hand-held deflection mechanism by the assistant or with the robotic grasping forceps and the perpendicular placement of the probe against the surface of the kidney or tumour.

Acquired ultrasound images can be streamed in real-time directly to the surgeon's DaVinci® console (Intuitive Surgical Inc., Sunnyvale, CA) using the multi-input display feature (TilePro) [47]. In contrast, traditional laparoscopic ultrasound probes have been considered to be slightly more challenging as they require re-adjustment, and the help of an assistant in comparison to the autonomy offered with the robotic forceps-adapted probe [48] and may be challenging to apply perpendicular to the surface of the kidney [46].

Only two of the studies included in this systematic review have specified the model of the ultrasound probe utilised. Sun et al. [19] used a BK Medical 8826 probe and Yang et al. [20] utilised a UST-5536-7.5 probe manufactured by Hitachi Aloka.

InUS performed by an expert (a urologist trained in ultrasound interpretation) can significantly reduce the chance of a PSM [43] and the adoption of InUS is not without its caveats. In terms of surgeon expertise and proficiency in using InUS, the authors propose that training on tissue or synthetic models as well as radiologist-led training programmes or the presence of a radiologist during “pilot” procedures can upskill surgeons with neither a high financial burden nor a steep learning curve foreseen. The second caveat identified by the authors is the cost of incorporating an ultrasound probe through initial investment, maintenance, and sterilisation. The financial aspect of InUS must be subject to a cost-benefit analysis and can be easily justified in high-volume oncology centres. A study comparing PN and RN in 2002 in the USA [49] concluded that there was an insignificant difference in cost between the two procedures while acknowledging that surgical instruments including InUS contributed towards the cost of PN.

The results of our systematic review demonstrate that there is a clear benefit in leveraging InUS to improve the rates of R0 resections, which in turn contributes towards the surgeon’s goal of achieving the “trifecta” in PN. InUS significantly enhances the management of RCC via precise localisation. This is especially true of intraparenchymal tumours, which, unlike predominantly exophytic masses, are challenging for even experienced surgeons to confidently locate and excise en masse. Additionally, InUS aids with resection planning and can potentially minimise warm ischaemia time while improving oncological outcomes.

Limitations

​​The studies included in this systematic review have provided valuable insight into the utility of InUS, yet it is crucial to acknowledge the limitations.

Firstly, there is a lack of availability of randomised controlled studies. The authors acknowledge the significant and potentially unjustifiable ethical considerations in developing a randomised controlled trial, where opting not to utilise intraoperative ultrasound in centres where the technology is available may impact oncological outcomes and bring patients to avoidable harm.

Secondly, the authors recognise the difficulty in comparing tumour characteristics and surgical complexity between studies as a result of under-reporting of the complexity of the renal masses through RENAL and PADUA scores, respectively.

Finally, high-powered, multi-centre experiences in the use of InUS using a standardised data collection tool should be used, with parameters including aforementioned scores, proximity, and involvement of the collecting system, location of the tumour within the parenchyma, and transducer types. This will allow meaningful statistical analysis to determine if there is a statistical significance in tumour margin outcomes when using InUS depending on tumour location and characteristics.

## Conclusions

InUS can be employed to ensure complete clearance of resection margins macroscopically by improving tumour visualisation in challenging or uncertain locations, specifically endophytic tumours; thereby increasing the success rates of nephrectomies and potentially improving disease-free survival and overall survival by reducing the number of positive surgical margins as demonstrated by the results from this systematic review. Furthermore, InUS can also aid in radical nephrectomies when combined with Doppler imaging to locate renal vasculature in cases of inferior vena cava (IVC) invasion confirmed on pre-operative MRIs.

Future publications should include high-powered, multicentre studies comparing the utility of InUS in partial nephrectomies for RCC by grade as well as the use of InUS in the management of tumour thrombus within the IVC. This systematic review highlights the potential for hospitals and healthcare services to include ultrasound technology as a part of the “robotic package” for nephrectomies.
